# Superficial intraventricular surface siderosis brain

**DOI:** 10.1016/j.radcr.2022.08.022

**Published:** 2022-09-06

**Authors:** Edlira Harizi, Kledisa Shemsi, Ilir Ahmetgjekaj, Anusha Parisapogu, Keti Mamillo, Fjolla Hyseni, Simmy Lahori, Kampa Prathima, Chandalji Naik Banavath, Zaina Syed, Srikrishnan Pichuthirumalai, Juna Musa, Jasmine Saini, Arieta Hasani Alidema, Valon Vokshi, Mohammad Abubaker Siddique

**Affiliations:** a Department of Neurology, Regional Hospital Durres, Albania; b Regional Hospital Durres, Tirana, Albania; c University Clinical Center, Clinic of Radiology, Pristina, Kosovo; d Department of Infectious Diseases, Mayo Clinic, Rochester, NY, USA; e Department of Anesthesiology, University Medical Center of Tirana “Mother Teresa”, Tirana, Albania; f Department of Pediatrics, NYU Langone Health, New York, NY, USA; g Department of Radiology, Mayo Clinic, Rochester, NY, USA; h Lifetime Wellness Rx International Ltd, Bengaluru, India; i Guntur Medical College, Andhra Pradesh, India; j Biochemistry, Hunter College CUNY, New York, NY, USA; k Sakra World Hospital, Bengaluru, India; l Department of Endocrinology, Diabetes and Nutrition, Mayo Clinic, Rochester, NY, USA; m Department of Endocrinology, Mayo Clinic, Rochester, NY, USA; n University for Business and Technology (UBT), Pristina, Kosovo; o Department of Anesthesiology, University Clinical Center of Kosovo, Pristina, Kosovo; p North East Medical College & Hospital, Sylhet, Bangladesh

## Abstract

Superficial siderosis of the central nervous system is a chronic condition characterized by hemosiderin deposition in the brain and spinal cord. It's diagnosed by brain MRI. It can be caused by low-grade extravasation of blood into the subarachnoid space of the brain. There are 2 types of superficial siderosis cortical and infratentorial. Although asymptomatic in many cases; Cerebellar-predominant siderosis, a subtype of infratentorial, can affect hearing, gait, and even muscles. In this report, we present a case of a 51-year old female with complaints of hearing loss, unsteadiness in his lower limb, and spastic paresis. During MRI neuroimaging, we noticed findings of hypointensity areas within the brainstem and cerebellum, probably due to hemosiderin deposition. Based on the MRI findings, the patient was diagnosed with superficial siderosis. The patient was started on deferiprone and followed for the consecutive 18 months. Moderate improvement of the hearing loss and ataxia was noted while no change in muscle force. However, the repetitive MRI did not reveal any changes compared to the previous one.

## Introduction

Superficial siderosis (SS) of the central nervous system (CNS) is a chronic condition characterized by hemosiderin deposition in the brain (and spinal cord) subpial layers as a result of chronic or intermittent low-grade extravasation of blood into the subarachnoid space [1]. There are 2 different types of superficial siderosis:

Cortical SS—Superficial hemorrhage in the cortical sulci causes hemosiderin deposition on localized supratentorial cerebral convexities [3,4]. It is associated with cerebral amyloid angiopathy (CAA), an age-related disease of the cerebral vasculature caused by amyloid deposition within the walls of small cortical and leptomeningeal arteries [5]. Symptoms of this type may include episodic and focal neurological symptoms related to the affected brain regions [3,4].

Infratentorial SS—Brainstem and cerebellar-predominant SS, also known as infratentorial SS, Hemosiderin deposition occurs in the posterior fossa, brain stem, and spinal cord [5]. It is caused by recurrent or persistent subarachnoid bleeding with hemosiderin deposition in the subpial layers of posterior fossa structures [1]. Bleeding is frequently caused by pathologies of the dural or nerve roots, prior surgeries, or tumors [5]. It is characterized by gait ataxia, myelopathy, or hearing loss [1].

Superficial siderosis leads to progressive and irreversible neurological dysfunction [2]. The clinical course of SS is determined by the location and extent of hemosiderin deposition. Patients may also experience symptoms related to the underlying syndrome or condition that caused the siderosis. Others may be asymptomatic, with SS discovered incidentally during an unrelated imaging procedure [2]. Sensorineural hearing loss, cerebellar ataxia, and myelopathy are the classic symptoms of the condition [2]. The most common causes of chronic subarachnoid hemorrhage that lead to superficial siderosis include CNS tumors, head and neck trauma, and arteriovenous malformations, although an occult source is unidentified in over a third of cases [2]. Brain MR imaging is the investigation of choice for the diagnosis of SS [1]. The underlying etiology is frequently identified by syndromic clinical symptoms or findings on diagnostic brain MRI.

## Case presentation

A 51 years of age patient arrives at our clinic with complaints of hearing loss, unsteadiness in his lower limb, and spastic paresis. The complaints started 14 years prior with slow progression over the consecutive years. General physical examination revealed no significant changes. Further investigations for a history of trauma, surgery, and tumors were absent. No delayed recall score was present on mental examination. On neurological examination, sensorineural hearing loss, a weak finger to nose test, and reduced force of the lower limbs. Laboratory tests were within range, including blood and urine analyses, blood coagulation, liver and kidney functions, thyroid tests, and vitamin concentrations. In addition, the patient's antinuclear antibody, anti‑O antibodies, erythrocyte sedimentation rate, and tumor markers were all normal. During MRI neuroimaging, we noticed findings of hypointensity areas within the brainstem and cerebellum, probably due to hemosiderin deposition. Based on the MRI findings, the patient is diagnosed with Superficial Siderosis. The patient is started on deferiprone and followed for the consecutive 18 months. Moderate improvement of the hearing loss and ataxia, while no change in muscle force. However, the repetitive MRI did not reveal any changes compared to the previous one.

## Discussion

Superficial siderosis consists of hemosiderin deposition within the CNS inner layers (brain or spinal cord). It can be associated with any form of CNS hemorrhage including SAH, SDH, and primary intraparenchymal/intraventricular hemorrhage [6]. According to a case report published in January 2007, for unknown reasons, males are affected more than females in the ratio of 2:1 across all the etiologies. The average age of presentation is in the fifth or sixth decade although the range of presentation can be from birth to adulthood [2]

The pathologic changes associated with SS have been previously described. Macroscopically a dark, brownish discoloration has been present, while microscopically extensive hemosiderin has been described. In addition, thickened leptomeninges, neuronal loss of various degrees, reactive gliosis, and demyelination have been found [7].

Throughout our review, the presence of a recurrent hemorrhage is a must for SS development. However, there have been some etiological groups of pathologies causing the hemorrhage. Among the well-known causes, SS is most commonly caused by a spinal dural defect in 47% of cases. Tumors including ependymoma, oligodendroglioma, pineocytoma, and paraganglioma are responsible for 35% of SS. CNS tumor could be one of the sources of bleeding, both pre- and postoperatory. Vascular malformations including cavernous angioma, arteriovenous malformation, and aneurysm are responsible for 18% of cases of SS [8,9].

The SS is often characterized by a triad of symptoms, sensorineural hearing loss (95%), cerebellar ataxia (88%), and pyramidal signs (76%) [9]. Depending on the location, other symptoms may be reported. The interval between symptoms and the preceding disorder may take years [10].

**Fig. 1:**
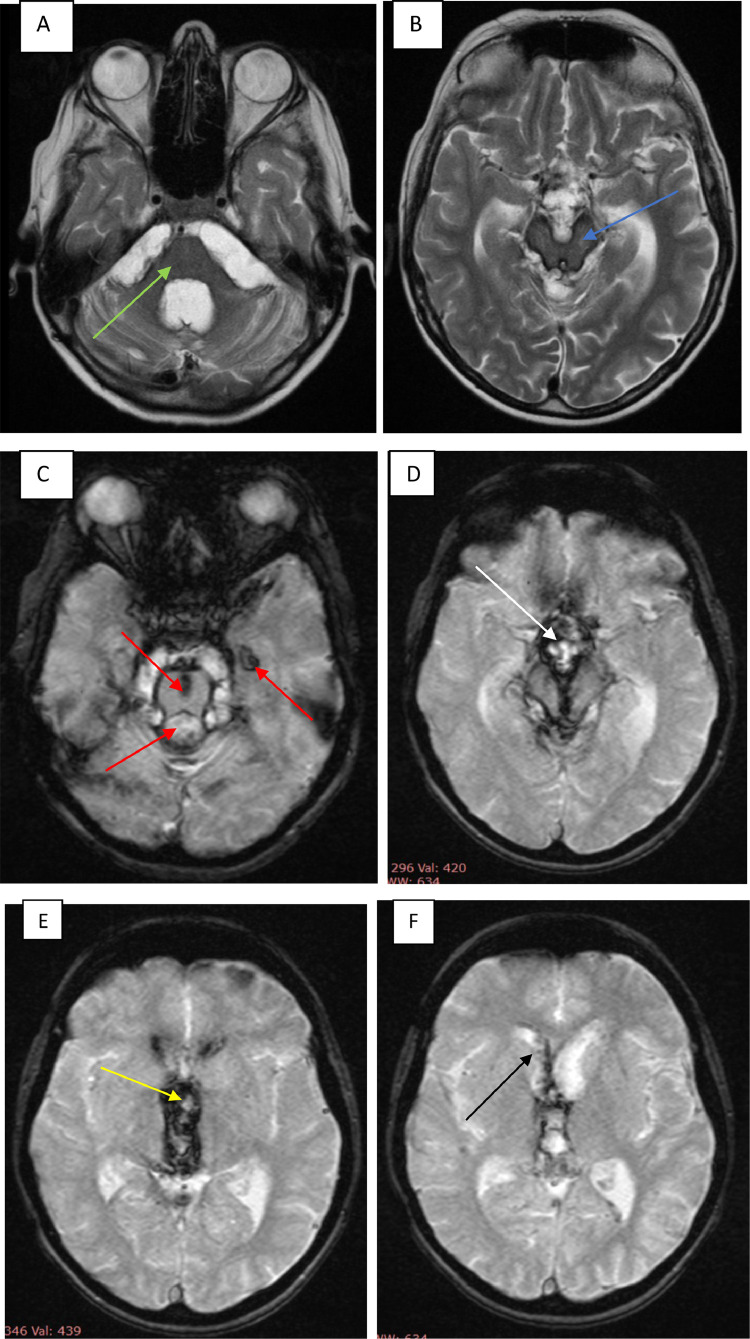
Typical magnetic resonance imaging (MRI) findings in superficial intraventricular surface siderosis. T2-weighted axial images present hemosiderindeposits along the cerebellar peduncles, around the IV ventricle (A) (green arrow) mesencephalon) (B) (blue arrow). T2-weighted images GRE presents hemosiderin deposits along the peripontine cistern, pons, Sylvi Aquedactus, tentorium, temporal horn of the left lateral ventricle (C) (red arrows), mesencephalon, suprasellar pons cistern (D) (white arrow), III ventricle (E) (yellow arrow), and frontal horns (F) (black arrow).

Superficial siderosis is considered a secondary effect instead of a real diagnosis. Therefore, the clinical symptoms and MRI findings may not correspond. MRI diagnosis can be made in the absence of symptoms [11], assuming MRI is the investigation of choice in relation to CSF fluid ferritin investigation (Fig. 1).

Ideally, the treatment of the cause would interrupt the SS development. Some chelation therapies have been attempted. Deferiprone can be used safely with closed monitoring of possible side effects until the discovery of a new and effective option for the treatment of superficial siderosis [10].

## Patient consent

Written informed consent for the publication of this case report was obtained from the patient.
